# Revisiting the Relative Age Effect From a Multidisciplinary Perspective in Youth Basketball: A Bayesian Analysis

**DOI:** 10.3389/fspor.2020.581845

**Published:** 2021-02-02

**Authors:** Carlos Eduardo Gonçalves, Humberto Moreira Carvalho

**Affiliations:** ^1^Faculty of Sport Sciences, University of Coimbra, Coimbra, Portugal; ^2^School of Sports, Federal University of Santa Catarina, Florianopolis, Brazil

**Keywords:** relative age effect, youth sport, selection, Bayesian analysis, multidiscipinary approach

## Abstract

Relative age effect (RAE) is considered to bias the selection of young athletes and a cause of exclusion of many participants. The goal of the study was to unveil the effects of the birth quarter on physical performances and a set of psychological constructs in the age groups corresponding to the specialization years. A set of surveys with cross-sectional data collected from 2015 to 2019 in youth basketball was used. Three hundred and twenty-seven Brazilian players (127 females, 100 males), mean age 14.0 years, participated in the study. Counter-movement jump, line-drill, yoyo intermittent test, achievement goals, motivation for deliberate practice, and enjoyment were measured. Bayesian multilevel regression was performed. RAE was observed but its advantages did not persist and did not differentiate the players in the variables under scrutiny. The only predictor of athletic and psychological outcomes was chronological age. The initial advantage that triggered the coaches' decision to select individual players disappears during the specialization years. Coaches must overcome the superficial observation of young athletes based only on age groups and actual performances, avoiding hasty decisions that, unlike RAE, last in time and cannot be reversed.

## Introduction

The last decade witnessed a plethora of studies on talent identification and development, aiming at identifying accurate predictors of adult elite performance (for a review, Sarmento et al., 2018). The first common characteristic of these studies is their utilitarian ideology. Starting with measurements and assessments at different stages of childhood and adolescence, researchers try to discriminate the variables that can help coaches and managers to select or deselect athletes. The premise is that sport specialization is a necessary condition for performance and expertise development, the theoretical ground being the deliberate practice theory (Ericsson et al., 1993). The corollary is that specialization must start in childhood and deliver the best possible athlete at the end of the process, preferably under the guidance of qualified coaches.

The second common characteristic is the persistent inconsistency of methods, results, and interpretations of the results. As Zuber et al. (2016) put it, “it is simply not possible to consider the entire person-environment system empirically in holistic terms.”

But if sports training is a behavioral praxis and is highly dependent on the multiplicity of contexts, the irruption of the biologic genotype occurs through the constructs of maturation and Relative Age Effect (RAE). The two phenomena are not to be confounded, but they are considered as an important source of biases in the decision-making process regarding selection or de-selection of young athletes, both male and female, and is transversal to multiple sports (Kirkendall, 2014; Hancock, 2017; Jones et al., 2018). However, unlike RAE, maturity status remains more difficult to assess in an exact manner (Koziel and Malina, 2018) as it requires a certain level of expertise in the field.

As the contemporary paradigm about sport specialization sets the beginning of training in a single sport at 5–6 years of age (Lovell et al, 2015; Müller et al, 2018), it means that the time for engagement, sampling, and performance covers childhood, adolescence, and the early stages of adulthood. According to Patel et al. (2019), it is precisely around 6 years that the RAE bias starts to occur. The phenomenon reflects a decision made by a coach at a certain stage of the participant's career. The negative effects of that decision would be the exclusion of potential talents just because they were chronologically younger by the time of selection.

Contrasting results about the eventual persistence of the bias in adult sport, depending on sport and contexts of competition have been noted (Werneck et al., 2016; Johnson et al., 2017; Haycraft et al., 2018; Rubajczyk and Rokita, 2018; Lupo et al., 2019; Kelly et al., 2020). However, the purpose of the present study is not to document the survival of the RAE during the path to expertise, but to investigate if the decision made by the coach when selecting athletes born in the first quarter of the year has effective consequences for the athletes' performance. Furthermore, the analysis was extended to all quarters of the year, encompassing all the possible effects originated by the date of birth.

Basketball performance is influenced by physical, physiological, and behavioral determinants (Carvalho et al., 2018). Particularly with young players, proper interpretations of body dimensions, functions, and behaviors need to adjust for the influence of growth and maturity status (Carvalho et al., 2018; Armstrong, 2019). Our goal here was to unveil the effects of the birth quarter on physical performances and a set of psychological constructs in the age groups corresponding to the specialization years. In previous studies we used an interdisciplinary perspective (Carvalho et al., 2018; Soares et al., 2020), combining measures of physical performance with psychological assessment through the use of questionnaires. Hence, the dependent variables are related to speed, strength, and intermittent endurance (Stojanovic et al., 2018), but also to achievement goals (Helmreich et al., 1978), motivation for deliberate practice (de Bruin et al., 2007) and enjoyment (Wiersma, 2001). It seems plausible that the physical and psychological advantages seen by the coach at the time of selection are translated into better athletic performances and psychosocial adaptations.

We choose the Brazilian context because traditionally participation in organized youth sport is possible only through a process of tryouts performed at the beginning of the season when coaches decide those who are accepted in the team and those who are excluded. The ages under observation (10–18 years) correspond to the sampling and specialization stages defined in the current development models (Côté and Vierimaa, 2014).

In sports sciences, samples are often non-normal and imbalanced, and the sources of variation are multiple and nested in hierarchical orders of influence. On the other hand, the combination of physiological and behavioral measures can be potentially noisy and lead to biased and unreliable inferences. The present analytical approach is deemed to deal with imbalanced samples and missing values in some outcomes. As an alternative to the exclusion of athletes, we modeled our data using a Bayesian multilevel modeling framework.

The purposes of the present study are: to estimate the effects of the birth quarter, combined with gender, age group, maturity status, and age of start of organized training on (a) the performance in counter-movement jump, line drill, and yoyo, and (b) on measures of achievement goals, motivation to deliberate practice and enjoyment among basketball players aged 10–18 years.

## Methods

### Data

The data in this study was assembled by combining surveys with cross-sectional data collected from 2015 to 2019 in youth basketball. We considered observations from youth female and male teams in the São Paulo, Santa Catarina, and Rio Grande do Sul basketball federations: These federations are affiliated in the Confederação Brasileira de Basketball (Brazilian Basketball Confederation). The sample considered 327 Brazilian adolescent basketball players aged 14.0 (1.7) years, on average, with a range between 9.5 and 17.9 years. The sample included 127 female and 200 male players. The players participated in regular training sessions (3–5 sessions; 270–450 min per week) in their clubs. The competitive seasons typically run between February/March until November/December, including about 20–30 games per season. We grouped players as under-11, under-13, under-15, and under-17, assuming a 2-year range, which is the most common competitive age group in Brazilian youth sports.

Ethical approval was obtained from the authors' institutional ethics committee. All participants were informed about the nature of the survey, that participation was voluntary, and that they could withdraw from the study at any time. All participants and their parents or legal guardians provided written informed consent.

### Procedures

Chronological age was calculated by subtracting birth date from the day of testing to the nearest 0.1 year. Stature was measured with a portable stadiometer (Seca model 206, Hanover, MD, USA) to the nearest 0.1 cm. Body mass was measured with a calibrated portable balance (Seca model 770, Hanover, MD, USA) to the nearest 0.1 kg. Reliability estimates for the observer are published elsewhere (Soares et al., 2020). Considering the month of birth, we grouped players as follows: 1st quarter (*n* = 111), when a player was born in January, February, or March; 2nd quarter (*n* = 90), when a player was born in April, May, or June; 3rd quarter (*n* = 61), when a player was born in July, August, or September; 4th quarter (*n* = 65), when a player was born in October, November, or December.

The assessment of biological maturation is often overlooked. In this study maturity status was estimated based on the gender-specific maturity offset protocol (Moore et al., 2015). The offset equations predict time before or after PHV based on chronological age and stature. Then it is possible to estimate each player's age at PHV. Hence, the offset equations attempt to provide an estimation of timing (i.e., the age at which a given pubertal milestone is reached). Often the interest and interpretations lie on information about tempo, i.e., the rate of within-person progression through maturation stages. Hence, we compared the estimates of timing obtained with the offset equations against the population references based on meta-analysis estimations, providing an interpretation of between-individuals variation in maturity status. In particular, we contrasted the players' estimated age at PHV against a gender-specific reference age at PHV derived from a meta-analysis of longitudinal growth studies (Malina et al., 1988). The reference age at PHV was 11.9 (90% credible interval: 11.8, 12.0) years and 13.9 (90% credible interval; 13.8, 14.0) years for girls and boys, respectively (Lima et al., 2020). Then we classified players as follows: early maturers (*n* = 139), when estimated age at PHV was lower than the gender-specific reference age at PHV by more than 6 months; average matures (*n* = 162) when players' estimated age at PHV was within plus/minus 6 months of the gender-specific age at PHV; late matures (*n* = 26), when estimated age at PHV was higher than the gender-specific reference age at PHV by more than 6 months. Nevertheless, we assume the limitations of the maturity offset protocol (Carvalho et al., 2018), particularly at the extremes of the observed age range where bias may be more significant (Koziel and Malina, 2018).

The onset in basketball was considered as the self-reported age when athletes started formal training and competition in basketball, under the supervision of a coach within a youth basketball program registered in the state basketball federation, and with no participation in practice and competition in other organized sport. Then the onset of deliberate basketball practice was related to two biological maturation milestones, the age of onset of the pubertal growth spurt and the age at PHV. Again, the biological milestones were estimated based on data from longitudinal growth studies (Malina et al., 1988). Details about the Bayesian multilevel modeling to perform a meta-analysis and derive the estimates are provided elsewhere. The reference age of the pubertal growth spurt onset was 9.4 (90% credible interval: 9.0, 9.8) years and 11.1 (90% credible interval: 10.8, 11.5) years for girls and boys, respectively. We grouped the players by the onset of deliberate basketball practice as follows: pre-puberty deliberate basketball practice onset (*n* = 155), the players who started practice before the reference age of pubertal growth spurt onset; mid-puberty deliberate basketball practice onset (*n* = 135), the players starting practice between the reference ages of pubertal growth spurt onset age and at PHV; late-puberty deliberate basketball practice onset (*n* = 37), the players starting practice after the reference age at PHV.

To describe players functional capacities we used the vertical jump with countermovement (Bosco et al., 1983), a short-term maximal running protocol, the line drill test (Semenick, 1990; Carvalho et al., 2017) and intermittent endurance test, the yo-yo intermittent recovery level 1 test (yo-yo IR1) (Bangsbo, 1994). Details about the functional performance procedures and reliability estimates are available elsewhere (Carvalho et al., 2018, 2019; Soares et al., 2020).

The psychological factors were assessed using the deliberate practice motivation questionnaire (de Bruin et al., 2008), the work and family orientation questionnaire (Helmreich et al., 1978), and the sources of enjoyment in youth sport questionnaire (Wiersma, 2001). It is composed by 28 items responded in a five-point Likert scale (1 = not at all, 2 = a little, 3 = not sure, 4 = yes, and 5 = very much), assessing, in the Portuguese version, the *s* (four items). We used an adapted version for basketball, translated and validated to Portuguese (Gonçalves et al., 2011) of the deliberate practice motivation questionnaire, originally designed for chess (de Bruin et al., 2007, 2008). The reliability of the adapted Portuguese version has been reported with data in youth basketball from the same age range of the present study elsewhere (Gonçalves et al., 2011). Similar to our previous observations in youth basketball (Gonçalves et al., 2011; Carvalho et al., 2018; Soares et al., 2020), we used the last three subscales of achievement from the work and family orientation questionnaire (Helmreich et al., 1978). Subscales considered were work (the desire to face challenging tasks the desire to practice and perform well), mastery (the desire to face challenging tasks), and competitiveness (the desire to be better when compared to others). For enjoyment we used the Portuguese version translated and validated by Santos and Gonçalves (2012), considering five subscales: positive parental involvement, self-referenced competencies, other-referenced competencies and recognition, effort expenditure and affiliation with peers.

### Statistical Analysis

#### Modeling Approach

Often, sports science survey samples are non-representative and imbalanced, there are hierarchical sources of variation or cross-classified nesting, and physiological and behavioral measures can be noisy measures that can potentially lead to biased and unreliable inferences. The present data is imbalanced and there are missing values in some outcomes. As an alternative to excluding players, we modeled our data using Bayesian multilevel regression and poststratification (Gelman and Little, 1997). The technique allows us to estimate the outcomes in small groups using varying effects for individual-level predictors such as gender, age group, maturity status, or onset of deliberate practice, that take on multiple levels in the data (Kennedy and Gelman, 2020) In the second part of the method, we use the multilevel model estimates to predict the players' outcomes for groups defined in a post-stratification dataset (i.e., gender, birth quarter, age group, maturity status, and the onset of deliberate basketball practice). The post-stratification table has an observation corresponding to each group defined for all combinations of the variables included in the model. Since, in the present study, models included two gender levels, four birth quarter levels, four age-group levels, three maturity status levels, and three onsets of deliberate practice levels, the post-stratification table encompassed 288 rows (2 × 4 × 4 × 3 × 3), including the sample size, in each group. After predicting the outcome variable for each group, we aggregated estimates for gender (or other subgroup units) with the subgroup sample sizes. Hence, the method allows us to take full use of all available data. Lastly, we prefer Bayesian methods as it provides a natural approach to account for different sources of inferential uncertainty (Kennedy and Gelman, 2020).

#### Model Specification, Priors, and Computation

Initially, we standardized the outcomes for interpretative convenience and computational efficiency. Our initial multilevel models were fitted with the intend to reproduce the naïve perceptions of the coach when selecting the players for the team. This reflects the common contexts in youth sport, where most clubs rely on the coaches' “eye,” with little help of specialized scouts or a multidisciplinary staff. Hence, the individual player's outcome was estimated as a function of his/her gender, birth quarter, and age group (for player i, with indexes g, b, and a for gender, birth quarter, and age group). Also, we allowed players in each birth quarter to vary when grouped by age group:

(1) $$\begin{array}{l} y_{i}=\;\beta^{0}+\;\alpha_{g\left[i\right]}^{gender}+\alpha_{b\left[i\right]}^{birth\;quarter}+\alpha_{a\left[i\right]}^{age\;group}\\ \;+\alpha_{a\left[i\right],b\left[i\right]}^{age\;group.birth\;quarter} \end{array}$$

The terms after the intercept are modeled as group effects (also referred to as random effects) drawn from normal distributions with variances to be estimated from the data:

$\alpha_{g\left[i\right]}^{gender}\;\sim \;N\;(0,\;\sigma_{gender}^{2}$), for g = 1, 2.

$\alpha_{b\left[i\right]}^{birth\;quarter}\;\sim \;N\;(0,\;\sigma_{birth\;quarter}^{2}$), for b = 1, 2, 3, and 4.

$\alpha_{a\left[i\right]}^{age\;group}\;\sim \;N\;(0,\;\sigma_{age\;group}^{2}$), for a = 1, 2, 3, and 4.

$\alpha_{a\left[i\right],b\left[i\right]\;}^{age\;group.birth\;quarter}\;\sim \;N\;(0,\;\sigma_{age\;group,birth\;quarter}^{2}$), for a = 1, 2, 3, and 4, and b = 1, 2, 3, and 4.

We extended the initial multilevel models to adjust for potential influences of maturity status and the onset of deliberate basketball practice. Hence, the individual player's outcome was estimated as a function of his/her characteristics, i.e., gender, birth quarter, age group, maturity status, and the onset of deliberate basketball practice (for player i, with indexes g, b, a, m, and d for gender, birth quarter, age group, maturity status, and deliberate basketball practice, respectively). Again, we allowed players in each birth quarter to vary when grouped by age group:

(2) $$\begin{array}{l} y_{i}=\;\beta^{0}+\;\alpha_{g\left[i\right]}^{gender}+\alpha_{b\left[i\right]}^{birth\;quarter}+\alpha_{a\left[i\right]}^{age\;group}\;+\alpha_{m\left[i\right]}^{maturity\;status}\\ \;+\alpha_{d\left[i\right]}^{deliberate\;practice}+\alpha_{a\left[i\right],b\left[i\right]}^{age\;group.birth\;quarter} \end{array}$$

Again, the terms after the intercept are modeled as group effects drawn from normal distributions with variances to be estimated from the data:

$\alpha_{g\left[i\right]}^{gender}\;\sim \;N\;(0,\;\sigma_{gender}^{2}$), for g = 1, 2.

$\alpha_{b\left[i\right]}^{birth\;quarter}\;\sim \;N\;(0,\;\sigma_{birth\;quarter}^{2}$), for b = 1, 2, 3, and 4.

$\alpha_{a\left[i\right]}^{age\;group}\;\sim \;N\;(0,\;\sigma_{age\;group}^{2}$), for a = 1, 2, 3, and 4.

$\alpha_{m\left[i\right]}^{maturity\;status}\;\sim \;N\;(0,\;\sigma_{maturity\;status}^{2}$), for m = 1, 2, 3.

$\alpha_{d\left[i\right]}^{deliberate\;practice}\;\sim \;N\;(0,\;\sigma_{deliberate\;practice}^{2}$), for d = 1, 2, 3.

$\alpha_{a\left[i\right],b\left[i\right]\;}^{age\;group.birth\;quarter}\;\sim \;N\;(0,\;\sigma_{age\;group,birth\;quarter}^{2}$), for a = 1, 2, 3 and 4, and b = 1, 2, 3, and 4.

For computational and interpretation convenience, we standardized (*z*-score) the outcomes. We used weakly informative priors to regularize our estimates, normal priors (0, 2) for the population-level parameter (i.e., intercept), and normal priors (0, 1) for the group-level parameters. By standardizing the outcomes and using a normal (0, 1) prior for the parameters, we consider being unlikely for the effects among group-level estimates to be greater than one standard deviation of the outcome. We run four chains for 2,000 iterations with a warm-up length of 1,000 iterations for each model. We inspect the convergence of the chains with trace plots and inspected and validated the models using posterior predictive checks (Gelman et al., 2013). The models were implemented using Stan which was called using the “brms” package (Bürkner, 2017), in R statistical language (R Core Team, 2018). All data and R codes are available online at https://osf.io/9rbm5.

## Results

Descriptive statistics for the total sample are summarized in Table 1. Note that standardization was based on the mean and standard deviation for the total sample.

**Table 1:** Descriptive statistics [mean (standard deviation)] for the total sample of young female and male basketball players.

	**Mean (standard deviation)**
Chronological age, yrs	14.0 (1.7)
Maturity offset, yrs	1.25 (1.69)
Estimated age at peak height velocity, yrs	12.7 (0.8)
Onset of basketball deliberate practice, yrs	10.4 (2.3)
Stature, cm	169.0 (12.1)
Body mass, kg	63.8 (15.3)
Countermovement jump, cm	29.2 (6.3)
Line drill test, s	34.58 (2.83)
Yo-yo intermittent recovery test level-1, m	721.6 (417.8)
Performance score, *z*-score	0.11 (2.55)
Will to excel, 1–5	4.15 (0.77)
Will to compete, 1–5	4.40 (0.56)
Mastery, 1–5	4.20 (0.60)
Work, 1–5	4.48 (0.48)
Competitiveness, 1–5	3.77 0.71)
Self-referenced competencies, 1–5	4.50 (0.52)
Others-referenced competencies, 1–5	3.81 (0.81)
Effort expenditure, 1–5	4.73 (0.47)
Positive parental involvement, 1–5	4.42 (0.70)
Affiliation with peers, 1–5	4.51 (0.62)

The analysis of the sample distribution shows that those born in the first quarter of the year represent 33% of the total. If we add those born in the second quarter, we account for 61% of the total sample. So, the selection bias is real. On the other hand, late maturers represent only 8% of the total of the sample, with average maturers accounting for almost half of the athletes (Tables 2, 3).

**Table 2:** Distribution by month quarter across the age group sample of young female and male basketball players.

	**Under 11**		**Under 13**		**Under 15**		**Under 17**		**Total**
	**Female**	**Male**	**Female**	**Male**	**Female**	**Male**	**Female**	**Male**	
1st quarter	4	6	16	27	17	29	6	6	111
2nd quarter	3	3	16	25	9	20	4	10	90
3rd quarter	5	7	8	16	5	11	6	3	61
4th quarter	4	7	7	13	13	13	4	4	65
Total	16	23	47	81	44	73	20	23	327

**Table 3:** Distribution by month quarter across the estimated biological maturity status in the sample of young female and male basketball players.

	**Early maturers**		**Average maturers**		**Late maturers**		**Total**
	**Female**	**Male**	**Female**	**Male**	**Female**	**Male**	
1st quarter	3	41	33	27	7	0	111
2nd quarter	4	37	25	21	3	0	90
3rd quarter	1	20	19	15	4	2	61
4th quarter	5	28	14	8	9	1	65
Total	13	126	91	71	23	3	327

**Figure 1:**
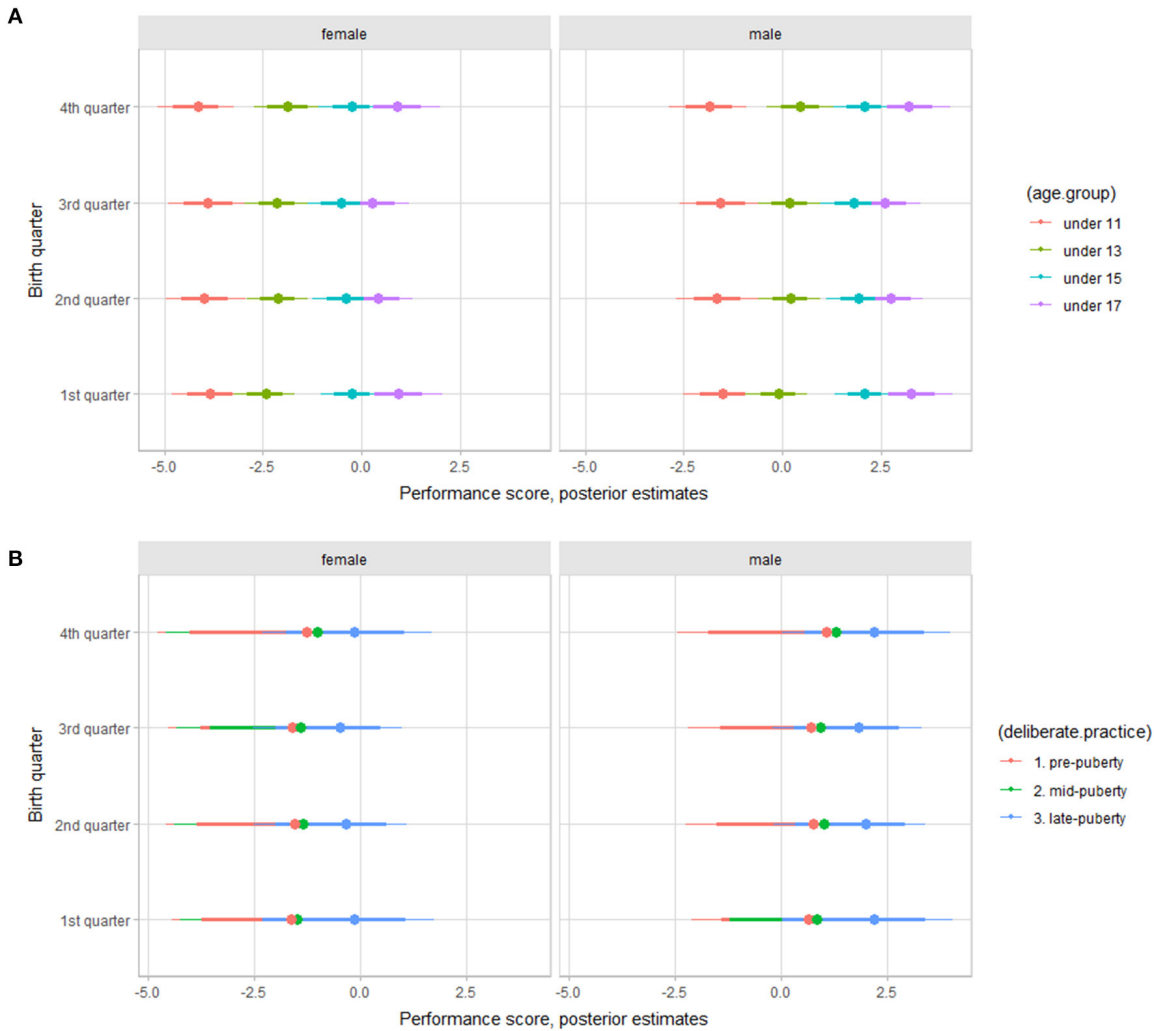
Posterior estimations for Performance score (*z*-score scale) by age group **(A)**, onset of deliberate basketball practice **(B)** in young female and male basketball players (67 and 90% credible intervals).

The results from the multilevel models considering the influence of gender, birth quarter, age group, maturity status, and the onset of deliberate basketball practice on the outcomes are summarized in Table 4. Given the extension of the results the plots of outcomes adjusted by birth quarter, separately gender and contrasting by age group, maturity status, or onset of deliberate basketball practice are available as supplementary material (https://osf.io/9rbm5). Here we present the plot of performance score, as an overall descriptor of players' athletic performance, examining the relative age effect separately by gender, and grouping by age group and onset of deliberate basketball practice in Figure 1. The adjusted estimates for athletic performance showed a similar pattern of RAE for both female and male athletes. Overall, there was a small, at best, trend of variation by birth quarter, as players born in the 1st and 4th trimesters showed better performance scores, particularly for the under 17 age group and for players with a late onset of deliberate basketball practice. Adjusted estimates showed that older players had a better athletic performance. Players with a late onset of deliberate basketball practice appeared to present slightly better athletic performance scores, however, uncertainty estimates were large.

**Table 4:** Multilevel regression models posterior estimations and 90% credible intervals of young basketball players by gender, age group, maturity status, onset of deliberate practice, birth quarter, and by the interaction of age group and birth quarter.

	**Population-level effects**	**Group-level effects**					
	**Intercept**	**Gender**	**Age group**	**Maturity status**	**Onset of deliberate practice**	**Birth quarter**	**Age group, birth quarter**
Countermovement jump	−0.13 (−1.05; 0.82)	**0.92 (0.43; 1.56)**	**0.72 (0.38; 1.18)**	0.28 (0.04; 0.61)	0.16 (0.01; 0.39)	0.12 (0.02; 0.27)	0.08 (0.01; 0.18)
Line drill test	0.13 (−0.73; 1.01)	**0.67 (0.22; 1.31)**	**0.96 (0.57; 1.44)**	0.18 (0.02; 0.45)	0.17 (0.02; 0.37)	0.12 (0.02; 0.27)	0.12 (0.02; 0.23)
Yo-yo intermittent recovery test level-1	−0.02 (−1.04; 0.95)	**0.82 (0.36; 1.42)**	**0.69 (0.35; 1.13)**	0.26 (0.04; 0.56)	**0.47 (0.17; 0.89)**	0.24 (0.04; 0.50)	**0.21 (0.05; 0.37)**
Performance score	−0.13 (−1.57; 1.29)	**1.34 (0.78; 2.01)**	**1.66 (1.13; 2.25)**	0.43 (0.06; 0.78)	0.38 (0.05; 0.81)	0.26 (0.03;0.57)	**0.41 (0.10; 0.72)**
Will to excel	−0.05 (−0.72; 0.62)	**0.60 (0.16; 1.22)**	0.16 (0.02; 0.34)	0.23 (0.03; 0.52)	0.28 (0.05; 0.63)	0.23 (0.04; 0.48)	0.09 (0.01; 0.20)
Will to compete	0.04 (−0.55; 0.65)	0.37 (0.03; 0.94)	0.28 (0.07; 0.57)	0.35 (0.08; 0.74)	0.25 (0.03; 0.58)	0.18 (0.02; 0.38)	**0.22 (0.09; 0.36)**
Mastery	0.01 (−0.65; 0.63)	0.57 (0.14; 1.17)	0.17 (0.02; 0.38)	0.27 (0.03; 0.60)	0.19 (0.02; 0.45)	0.17 (0.02; 0.45)	0.13 (0.02; 0.25)
Work	−0.03 (−0.68; 0.62)	0.53 (0.12; 1.15)	0.23 (0.04; 0.47)	0.25 (0.03; 0.62)	0.29 (0.06; 0.63)	0.23 (0.04; 0.50)	**0.23 (0.09; 0.38)**
Competitiveness	0.02 (−0.64; 0.70)	0.55 (0.13; 1.16)	0.26 (0.04; 0.55)	0.28 (0.03; 0.63)	0.33 (0.06; 0.71)	0.14 (0.02; 0.33)	0.13 (0.02; 0.27)
Self-referenced competencies	0.04 (−0.56; 0.63)	0.43 (0.06; 1.00)	0.15 (0.02; 0.33)	0.30 (0.05; 0.64)	0.31 (0.05; 0.67)	0.13 (0.02; 0.28)	0.14 (0.03; 0.26)
Others-referenced competencies	−0.08 (−0.76; 0.55)	0.37 (0.03; 0.95)	**0.39 (0.10; 0.78)**	0.28 (0.04; 0.62)	**0.44 (0.12; 0.88)**	0.17 (0.02; 0.39)	**0.20 (0.05; 0.34)**
Effort expenditure	−0.03 (−0.59; 0.56)	0.23 (0.03; 0.52)	0.40 (0.04; 1.00)	0.22 (0.02; 0.53)	0.25 (0.03; 0.60)	0.18 (0.02; 0.40)	**0.32 (0.18; 0.48)**
Positive parental involvement	−0.01 (−0.74; 0.65)	0.57 (0.14; 1.15)	0.32 (0.10; 0.61)	0.21 (0.02; 0.52)	0.39 (0.07; 0.82)	0.17 (0.02; 0.38)	0.16 (0.02; 0.30)
Affiliation with peers	−0.04 (−0.59; 0.50)	0.16 (0.02; 0.35)	0.39 (0.05; 0.93)	0.21 (0.02; 0.49)	0.34 (0.05; 0.75)	0.14 (0.02; 0.32)	**0.20 (0.05; 0.35)**

*Estimates in bold indicate substantial a variation at group-level on the outcome*.

**Figure 2:**
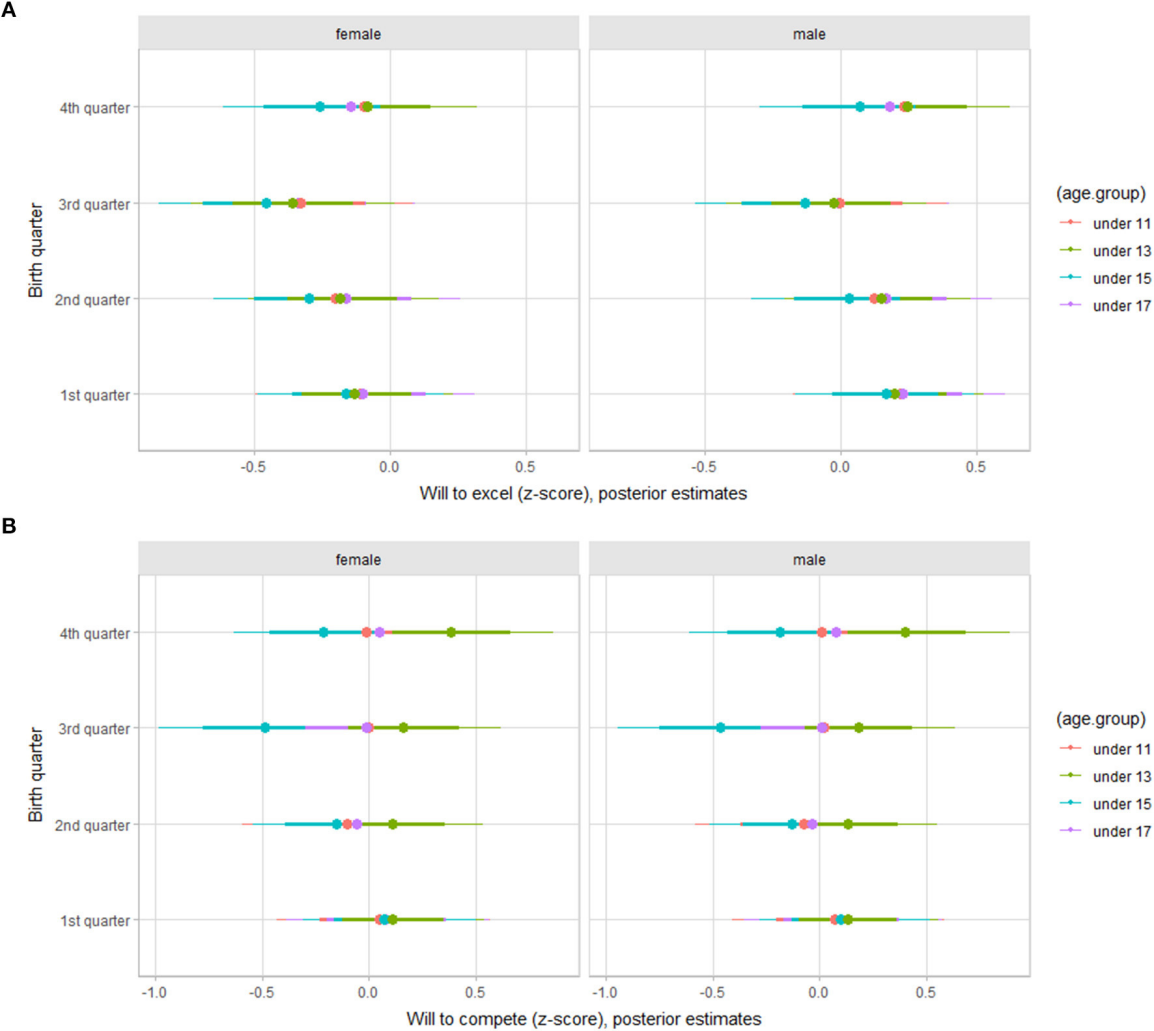
Posterior estimations for standardized scores for will to excel **(A)** and will to compete **(B)** by age group in young female and male basketball players (67 and 90% credible intervals).

**Figure 3:**
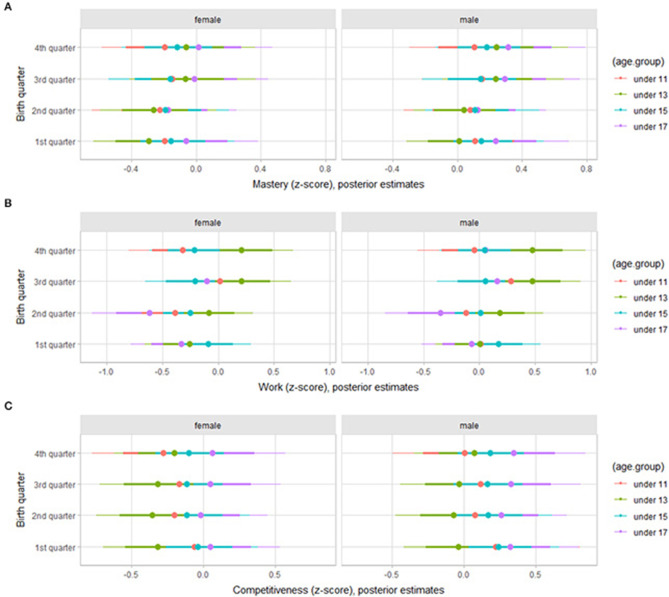
Posterior estimations for standardized scores for mastery **(A)**, work **(B)**, and competitiveness **(C)** by age group in young female and male basketball players (67 and 90% credible intervals).

Regarding the orientation to deliberate practice, there was a trend of lower values of will to compete for players born in the 3rd and 4th trimesters in the under 15 and 17 age groups (Figure 2). As for achievement goals, mastery, work, and competitiveness, there was a trend of higher values of work dimension for the under 13 age group players born in the 3rd and 4th trimesters, independent of gender (Figure 3).

**Figure 4:**
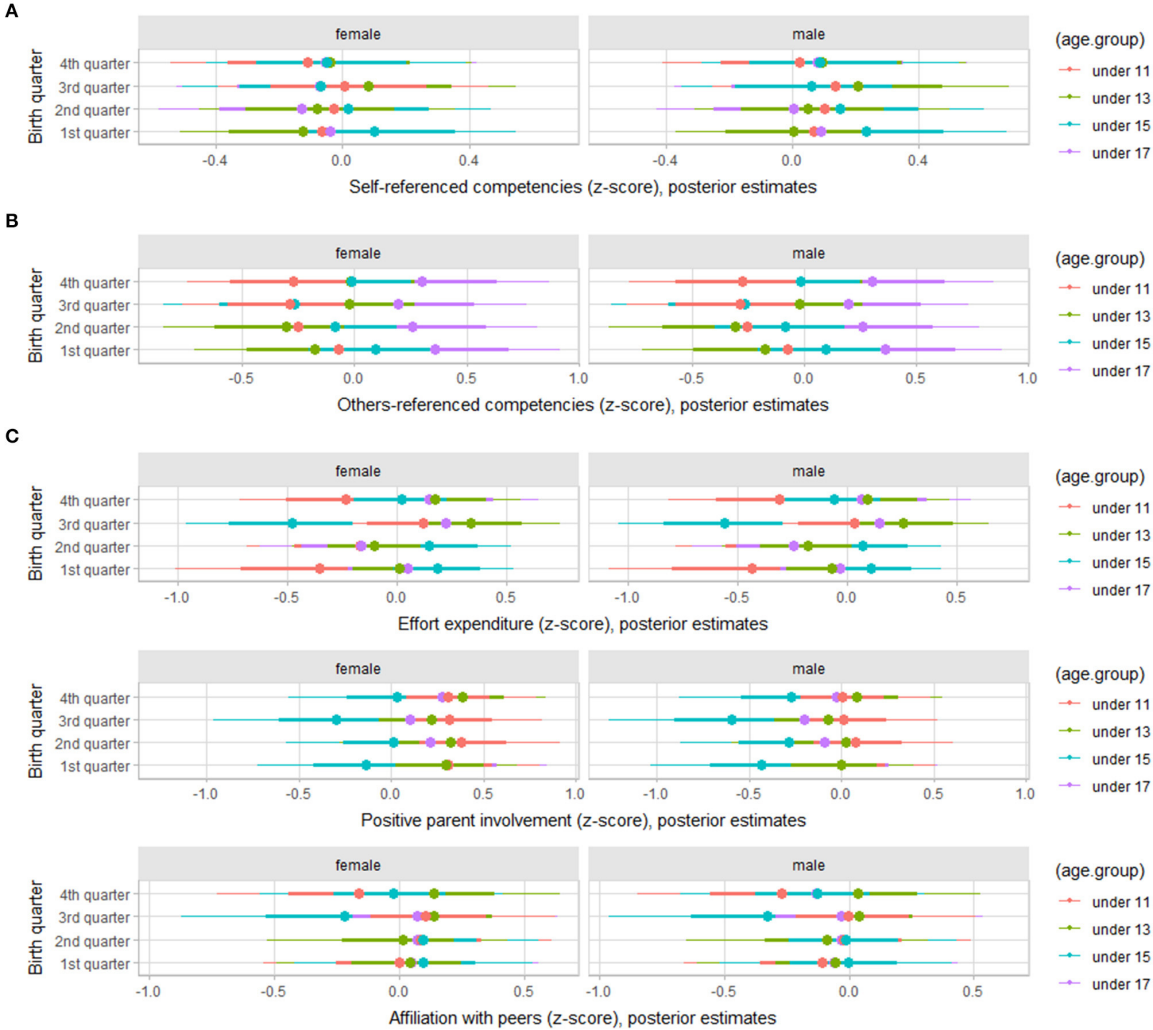
Posterior estimations for standardized scores for self-referenced competencies **(A)**, others-referenced competencies **(B)**, effort expenditure **(C)**, positive parental involvement **(D)**, and affiliation with peers **(E)** by age group in young female and male basketball players (67 and 90% credible intervals).

Regarding the sources of enjoyment, multilevel modeling and post-stratified estimates showed a substantial variation by birth trimester across age groups, particularly on the scores of others-referenced competencies of under 17 players (Figure 4). For self-referenced competencies, the under-15 players born in the 1st trimester showed higher scores. It is important to stress that there was no variation by gender on sources of enjoyment outcomes. For affiliation with peers, under-13 players born in the 4th trimester showed the higher scores, and under-15 players born in the 3rd quarter showed present the lowest scores. For effort expenditure, under-15 players showed contrasting scores between those born in the 1st and 2nd trimesters and those born in the 3rd and 4th trimesters; but the under-11 players showed consistently lower scores.

## Discussion

The purpose of the study was to estimate the effects of the birth quarter on a set of athletic and psychosocial variables, assembled through surveys with cross-sectional data, covering 5 years. The distribution of the sample shows an overrepresentation of players born in the first quarter of the year and of early- and average maturers. The utility of Bayesian methods and multilevel techniques with poststratification is evident, allowing us to overcome the distribution pitfalls and to estimate the outcomes in such small groups. To our knowledge, it is one of the few studies to perform this research design with the aforementioned statistical analysis (Kalén et al., 2020).

Our initial model is simpler and looks to reproduce the naïve perceptions of the coach when selecting the players for the team. In youth sport, most clubs rely on the coaches' “eye,” with little help from specialized scouts or a multidisciplinary staff. Thus, the coach makes a decision based on what he/she sees at the moment, e.g., the more capable athletes in the given situation, in that particular age group. Within the present sample, the RAE selection bias followed the trends reported in the literature (Arrieta et al., 2016; Hancock, 2017; Haycraft et al., 2018). Furthermore, the bias seems to be complemented by the overrepresentation of early- and average maturers, suggesting a homology between the RAE and the maturity status. But a close inspection of Table 2 reveals that the players born in the 4th quarter are early-maturers. The result is especially evident for male players, as the girls born in the 1st quarter and early-maturers are fewer than their male peers.

This reflects further evidence that RAE and maturity status are not to be confounded, although the present study suggests the phenomenological emergence of the “survival of the fittest” (Jones et al., 2018), as, at least for boys, those retained by the coaches are the older ones, chronologically and biologically. Hence, we added complexity to the second model by introducing maturity status and the onset of organized basketball practice as explanatory variables. The results do not seem to follow an established pattern, in line with the birth quarter or age group, but they express a common characteristic: being born in the 1st quarter does not necessarily present any advantage in all variables under scrutiny. Considering for athletic performance, achievement goals, or other-referenced competencies, the best scores are estimated to under-17 players, regardless of birth quarter and maturation, meaning that chronological age explains the results.

In most of the variables, the results by birth quarter and age group are difficult to interpret and probably reflect the specificities of the diverse contexts. The effects of biological maturation and accumulated experience in organized basketball did not account substantially for the interpretation of the outcomes. This may be partially explained by the trend of homogeneity in size, function, and experience among young athletes as results of the selection process in youth sports (Malina, 1994). It seems that the superficial look of the coach through the lens of the age group has no consequences in the long term. The lack of explanatory power of the variables included in the model corroborates the argument that during the sampling and specialization years (Côté and Vierimaa, 2014) the outcomes are more a consequence of the athletes' responses to the training loads and to the ecologies of practice than determined by a particular characteristic like the birth quarter, maturity status, or the year of engagement in basketball.

The assessment of athletic and psychosocial variables is important because sports training is a holistic intervention that cannot be separated through isolated and analytical approaches (Carvalho et al., 2018). Furthermore, there are studies (McCarthy et al., 2016; Cumming et al., 2018) pointing that younger athletes, later maturers, or born in the 4th quarter of the year, seem to display a psychological advantage. The present study shows that there are no differences in psychosocial constructs and, when they appear, are generally in favor of the chronologically older players.

There are conflicting reports about the persistence or reversal of RAE advantage in adult teams (McCarthy et al., 2016; Rubajczyk and Rokita, 2018; Lupo et al., 2019). The concern with RAE is linked to the fact that relatively young players are less likely to be selected into the organized sport system, but those who survive are more likely to transition to elite competitive levels.

But our study brings evidence that the initial advantage that triggered the decision to select individual players disappear much sooner, albeit when is too late for the coach to reverse the exclusion decision. The risk is to look for an archetype athlete with a theoretical existence belonging to all theories, as the product of an explanatory classification, one which is altogether similar to those of zoologists and botanists. These classifications give the illusion that scientists and coaches can explain and predict the future outcomes of the classified individuals, including their propensity to attain elite performances. But the reality is more prosaic and superficial cross-sectional evaluations do not translate often into vivid existences.

One final remark must be made about the gender issue. In the study, there are no apparent differences between boys and girls regarding the variables under-appreciation, and they seem to present the same pattern of results, in both athletic and psychosocial measures. This does not mean that there are no differences at all and that they must be coached in the same way.

The study has some limitations. It is confined to one sport and, although representing the local contexts of practice, it stills lacks critical dimension, namely regarding the balance between male and female athletes. We opted for a non-longitudinal design as the combination of surveys with cross-sectional data allowed us to model the data and estimate the players' outcomes for groups defined in a post-stratification dataset. But a longitudinal design would be also a suitable method to reach the same purposes. The study of multiple sports with larger samples, alongside the clear demarcation between RAE and maturity status, are future avenues for research.

## Conclusions

RAE has been consistently present in recent literature as selection bias and a factor of exclusion of participants and potential talents. Most studies have assessed the persistence or reversal of RAE among adult athletes. The present study shows that the initial advantage/disadvantage that was the cause of the decision to select or exclude disappears sooner than expected, as the athletes grow older and become fully immersed in the sport. Coaches must overcome the superficial observation of young athletes based only on age groups and actual performances and endorse a pragmatic realism, avoiding hasty decisions that, unlike RAE, last in time and cannot be reversed.

## Data Availability Statement

The original contributions presented in the study are publicly available. This data can be found at: [https://osf.io/9rbm5].

## Ethics Statement

The studies involving human participants were reviewed and approved by Ethics Committee of the Federal University of Santa Catarina. Written informed consent to participate in this study was provided by the participants' legal guardian/next of kin.

## Author Contributions

All authors contributed equally to the design, data collection, and writing of the paper.

## Conflict of Interest

The authors declare that the research was conducted in the absence of any commercial or financial relationships that could be construed as a potential conflict of interest.
